# Prevalence and management of depression among patients with chronic disease: A cross sectional study

**DOI:** 10.6026/973206300214297

**Published:** 2025-12-15

**Authors:** Ishani Samir Pandya, Shivangi Saxena, Mohammed Zakiullah Shareef, Urmi Jayesh Kothari, Shanmukha Koppolu, Kruthi Devaraj

**Affiliations:** 1 Vibrant Multispecialty Hospital, Gujarat, India; 2 Department of Psychiatry, Hampshire and Isle of Wight Healthcare NHS Foundation Trust, Isle of Wight, United Kingdom; 3 Department of Internal Medicine, Satyadev Hospital Anakapalle, Andhra Pradesh, India; 4 Department of Casualty, Bombay Hospital, Mumbai, India; 5 Department of Trauma and Orthopaedics, Barts NHS Trust, London, England, United Kingdom; 6 Department of Emergency Medicine, Manipal Hebbal, Karnataka, India

**Keywords:** Depression, chronic disease, prevalence, family medicine, primary care, management, collaborative care

## Abstract

Depression is a common comorbidity for patients with chronic health conditions, but its detection and treatment in the context of usual care is significantly inadequate. Therefore, it is of interest to determine the prevalence of depression and the management of depression among an adult population living with chronic health conditions attending outpatient services. A validated depression screening tool was administered to assess symptoms of depression, and management of depression was assessed with structured questionnaires. The findings revealed that depression was highly prevalent, and depression was frequently underdiagnosed and under-treated within the cohort, which highlights the need for screening for depression and applying the integrated care management model based on evidence-based guidelines into primary care contexts.

## Background:

Depression is a common comorbidity in chronic disease patients (prevalence estimates between 9 and 25%), often leading to an increased disease burden and progressive decline in outcomes [1]. In certain patients' populations with cardiovascular disorders, for example, the prevalence of depressive symptoms can increase as high as 59% [2]. The prevalence of depression in chronic pain conditions can also exceed 40%, demonstrating the duality between physical and mental health [3]. In the primary care setting, about one in ten patients report depression, and patients with multiple chronic comorbidities report this burden at notably higher rates [4]. The impact of depression on chronic disease management is more than the burden of disease states; an episode of depression has been shown to lead to worse quality of life, decreased adherence to medical therapies, poor engagement in patient self-care, and worse illness outcomes [5]. There is an increasing body of evidence to support integrated or collaborative care models, where mental health services are integrated within primary care, for improving depression-related outcomes and experience versus usual care alone [6]. There is also evidence to support a structured chronic disease management approach using tools like patient registries, care coordination, periodic follow up, multidisciplinary review, and all-encompassing approaches with evidence cited supporting improvements in adherence, reduced symptoms and enhanced patient reported outcomes [7]. These findings underscore the importance of proactive identification and management of depression in chronic disease populations. Therefore, it is of interest to evaluate the prevalence and impact of depression among patients with chronic diseases and to assess the effectiveness of integrated care approaches in improving both mental health and disease-related outcomes.

## Materials and Methods:

We conducted a cross-sectional study at a family medicine outpatient clinic in Andhra Pradesh. Adults (≥18 years) with at least one diagnosed chronic condition (e.g., diabetes, hypertension, chronic pain) were recruited consecutively over three months. Depression was screened using the Patient Health Questionnaire-9 (PHQ-9). We collected demographic data, type/duration of chronic disease, PHQ-9 scores and documented whether management included pharmacotherapy, psychotherapy, referral to psychiatry, or no treatment. Data were analyzed descriptively to estimate depression prevalence and summarize management patterns.

## Results:

Of the 200 patients with chronic disease who were surveyed, 70 (35%) screened positive for depression (PHQ-9 score ≥10), but only 30 (43%) had a documented diagnosis in the medical record. For those screening positive, 20 (29%) were receiving antidepressants, 10 (14%) were in counseling or psychotherapy, and only 5 (7%) received a referral to a psychiatrist, with 25 (35%) being untreated. Documentation of collaborative care components was limited: use of case registry in 7%, care coordination in 10%, and follow-up checks in 9% of depressed patients. The prevalence of depression was highest among patients with cardiovascular disease (40%) and those with a chronic disease diagnosis of greater than five years (38%). Overall, these data suggest there is a notable gap between screening for depression and having a diagnosis, managing depression in clinical practice, and implementation of collaborative care concepts in chronic disease patients. Table 1 (see PDF) shows the demographic and clinical characteristics of the 200 chronic disease patients, highlighting that depression prevalence was highest among those with cardiovascular disease and in patients with a longer duration (>5 years) of illness. Table 2 (see PDF) shows depression screening outcomes with documented medical record diagnoses, showing that more than half of patients who screened positive had no formal diagnosis recorded. Table 3 (see PDF) shows the types of depression management strategies used, indicating that a significant proportion (35%) of depressed patients received no treatment at all, with pharmacotherapy being the most common intervention. Table 4 (see PDF) shows and highlights the underutilization of collaborative care components in depression management for chronic disease patients, with care coordination being documented in only 10% of cases.

## Discussion:

Our study revealed a notably high prevalence of depression (35%) among patients with chronic diseases attending family medicine clinics. This rate is substantially higher than the prevalence typically reported in the general primary care population, which ranges from 10% to 20% in various studies [8]. Such findings align with prior evidence that chronic medical illnesses-such as diabetes, cardiovascular disease and chronic respiratory conditions-are strongly associated with increased risk for depression, likely due to a combination of biological, psychological and social stressors. Despite this elevated prevalence, we found that only 43% of individuals screening positive on the PHQ-9 had a documented diagnosis of depression in their medical records, indicating a significant underdiagnosis problem [9]. The gap indicates that potentially relevant depressive symptoms might be missed in the context of chronic disease management, as clinical contacts with patients primarily address chronic physical illness parameters. It also emphasizes the need for ongoing systematic screening protocols and clinician training to identify and recognize mental health symptoms as part of complex multimorbidity profiles [10]. The management of identified depression was also suboptimal. Less than one-third of patients who had a positive screen received evidence-based interventions, such as pharmacotherapy or structured psychotherapy, a small number were referred to specialized mental health services. This supports findings of previous studies reporting that depression is often under-treated in primary care, particularly in the presence of chronic physical illness [11]. Barriers may include limited consultation time, perceived stigma, and patient reluctance to engage in mental health treatment and resource restrictions at the level of the primary care practice. Additionally, the collaborative care components-such as the use of care managers, systematic treatment-to-target strategies and regular monitoring of outcomes-found in some clinics studying management of depression were rare in our sample clinics, despite strong evidence that these models are associated with better depression outcomes in primary care populations [12]. Better outcomes for both mental health and physical disease are achieved when chronic disease management frameworks also address mental illness, particularly when the interventions also consider [13]. These results illustrate the urgent need to incorporate standard depression recognition and evidence-based management strategies into the care of patients with chronic disease in family medicine. The implementation of collaborative care strategies may be able to close the treatment gap by ensuring timely diagnosis of treatment, consistent follow-up and organized multidisciplinary intervention [14]. In addition, addressing mental health as a part of chronic disease management will contain multiple benefits to overall patient outcomes given the bidirectional relationship between depression and chronic diseases, where unrecognized depression can deteriorate the disease's prognosis, decrease adherence to treatment and worsen treatment management [15]. Future studies should that will identify practical barriers to recognize and treat depression in family medicine and their impact on both mental and physical health outcome over time.

## Conclusion:

Depression is highly prevalent and often underdiagnosed in patients with chronic diseases. Routine screening and evidence-based management should be integrated into family medicine care. Collaborative care models can help address treatment gaps and improve both mental health and chronic disease outcomes.
